# Per-Vertebra Prediction of Future Osteoporotic Fractures from Routine Computed Tomography Using a Two-Stage Machine Learning Framework

**DOI:** 10.3390/medicina62081518

**Published:** 2026-08-06

**Authors:** Kirill Riazanovskiy, Dāvids Orlovs, Jekaterina Stepanova, Victor Sineglazov, Ardis Platkajis, Olena Chumachenko

**Affiliations:** 1 Department of Artificial Intelligence, Institute for Applied System Analysis, National Technical University of Ukraine “Igor Sikorsky Kyiv Polytechnic Institute” (KPI), 03056 Kyiv, Ukraine; svm@kai.edu.ua (V.S.); chumachenko.o.i.-ai@edu.kpi.ua (O.C.); 2 Department of Radiology, Faculty of Medicine, Rīgas Stradiņa Universitāte (RSU), LV-1007 Rīga, Latvia; davids.orlovs@rsu.lv (D.O.); jekaterina.stepanova@rsu.lv (J.S.); ardis.platkajis@rsu.lv (A.P.); 3 Institute of Diagnostic Radiology, Pauls Stradiņš Clinical University Hospital (PSKUS), LV-1002 Rīga, Latvia

**Keywords:** vertebral fracture, osteoporosis, computed tomography, quantitative imaging biomarkers, radiomics, machine learning, fracture-risk prediction, opportunistic CT, conditional logistic regression, expected calibration error

## Abstract

*Background and Objectives:* Osteoporotic vertebral compression fractures affect approximately one in four postmenopausal women and carry substantial morbidity, yet established clinical tools such as dual-energy X-ray absorptiometry (DXA) provide only patient-level risk and do not identify which specific vertebra is most likely to fail. Computed tomography (CT) acquired for unrelated indications is the most widely available three-dimensional substrate for opportunistic screening, but published machine learning models for vertebral fracture risk almost universally operate at the patient level. The present study aimed to develop and rigorously validate a per-vertebra prediction pipeline applicable to both routine clinical lumbar-spine CT and opportunistic abdominal CT, both acquired for indications unrelated to osteoporosis screening. *Materials and Methods:* Two independent retrospective cohorts were assembled from a single academic centre: a routine clinical lumbar-spine CT cohort of 106 patients yielding 478 evaluable vertebrae, and a routine abdominal CT cohort of 126 patients yielding 589 evaluable vertebrae. Vertebral bodies were segmented automatically with TotalSegmentator v2 and the trabecular core isolated by morphological erosion. A panel of 505 quantitative imaging biomarkers compliant with Image Biomarker Standardisation Initiative recommendations was extracted, covering trabecular density, vertebral morphometry, classical texture, trabecular network architecture, sub-endplate vulnerability, low-density topology, radial heterogeneity and adjacent muscle quality. Within-patient feature engineering expanded the input pool to 1293 contextual descriptors. Three model families were evaluated under fully nested leave-one-patient-out cross-validation: ElasticNet logistic regression, a softmax-ranking approximation of conditional logistic regression, and a Two-Stage model combining a patient-level fragility score with a within-patient vertebral outlier score. Patient-level bootstrap resampling (2000 iterations) was used to obtain 95% confidence intervals. *Results:* On routine clinical lumbar-spine CT the Two-Stage model achieved a per-vertebra AUC of 0.750 (95% CI 0.704 to 0.795), an F1 of 0.549, a within-patient concordance index of 0.693, an expected calibration error of 0.044, and Hit@3 of 0.934. It was the only model evaluated that returned calibrated probabilities; the softmax-ranking and ElasticNet baselines gave expected calibration errors of 0.232 and 0.218 respectively. On opportunistic abdominal CT, the softmax-ranking model gave AUC 0.672 (95% CI 0.615 to 0.727). Selected biomarkers were dominated by regional trabecular density and trabecular network architecture; a stable core of lumbar features entered the model in 100% of cross-validation folds, indicating high reproducibility. The closest prior per-vertebra CT-based predictor in primary, non-surgical patients (Muehlematter and colleagues, 58-patient cohort) reported a per-vertebra AUC of 0.64, which is one of several reference points for the present results. Ten methodological variants and sensitivity analyses, including rank fusion, internal tissue normalisation and additional biomechanical features, did not provide statistically significant gains, indicating that the binding constraint at this sample size is data volume rather than methodology. *Conclusions:* A two-stage decomposition that separates systemic skeletal fragility from within-patient vertebral outlier status produces well-calibrated per-vertebra fracture-risk estimates from routine clinical lumbar spine CT and was the only model evaluated to do so, which is what permits a per-vertebra output to be reported as an absolute risk rather than as an ordering alone; a within-patient ranking model is preferable for opportunistic abdominal CT. The discrimination advantage of the decomposition over that baseline is numerical and consistent but not statistically established at this sample size, and the work is presented as a transparent and reproducible single-centre benchmark for the still under-developed per-vertebra prediction task. Its clearest near-term value is opportunistic, namely flagging elevated per-vertebra fracture risk on CTs already acquired for unrelated indications without additional radiation, cost or a dedicated densitometric study. External multi-centre validation is the necessary next step.

## 1. Introduction

Osteoporotic fragility fractures of the spine are the most common skeletal complication of advancing age. Vertebral compression fractures alone affect roughly one in four postmenopausal women, are associated with an age-adjusted one-year mortality hazard ratio above two, and carry a lifetime direct-cost burden comparable to that of hip fracture [1,2,3]. Despite this clinical importance, the majority of vertebral fractures are clinically silent and only a minority are diagnosed prospectively; more than two thirds are discovered incidentally on imaging acquired for unrelated reasons [4,5].

Dual-energy X-ray absorptiometry (DXA) remains the guideline-recommended tool for fracture-risk stratification and underpins instruments such as FRAX, but it has two well-documented limitations in the spine. First, its two-dimensional projection geometry summates cortical, trabecular and posterior-element bone, so segmental osteoporotic rarefaction is frequently masked by overlying osteophytes and other vertebral pathology. Second, a DXA T-score is by construction a patient-level summary and cannot indicate which specific vertebral level along L1 to L5 is the weakest link [6,7]. The latter limitation matters clinically because vertebroplasty and balloon kyphoplasty are segmental procedures performed on individual levels, and because any level-targeted strategy, whether imaging surveillance or a segmental intervention, presupposes that a single at-risk vertebra can be identified in advance; segmental interventions themselves remain reserved for vertebrae that have already fractured [8]. Phantomless trabecular attenuation measured on routine clinical abdominal or thoracic CT has been shown to track DXA areal bone mineral density with Pearson correlation coefficients in the 0.5 to 0.8 range, and to discriminate osteoporosis at attenuation thresholds in the 90 to 110 Hounsfield-unit range with sensitivity of 60 to 75% and specificity of 80 to 90% [9,10,11], which establishes routine clinical CT as a quantitative substrate independent of DXA.

Computed tomography (CT) provides a complementary three-dimensional substrate. Phantomless attenuation measurements made on routine abdominal or thoracic CT correlate strongly with DXA areal bone mineral density and predict subsequent osteoporotic fractures, even when extracted from images acquired for other indications [9,10,12,13,14]. Modern deep learning segmentation, in particular self-configuring three-dimensional architectures based on nnU-Net [15], has made automated per-vertebra analysis tractable at scale [16], and the past decade has seen a rapid expansion in CT-based osteoporosis research. Patient-level prediction studies now report receiver-operating-characteristic areas under the curve of 0.71 to 0.84 using single attenuation measurements [10,12], vertebral compressive strength derived from finite-element analysis [17], end-to-end deep neural networks trained on raw image volumes [18,19], level-specific volumetric bone mineral density thresholds [20], and combinations of trabecular texture with paraspinal muscle quality [21], radiomic and habitat-based models [22,23], osteosarcopenia association analyses [24] and convolutional networks on cropped vertebrae [25]. A pooled meta-analytic estimate across 79 studies and 162 models places the area under the receiver-operating-characteristic curve for vertebral fracture prediction near 0.82 [26,27]. Several studies have also addressed the closely related but clinically distinct problem of secondary fracture after augmentation [28,29,30].

Two methodological gaps in the per-vertebra fracture prediction literature remain, alongside one unmet clinical need that is a scope choice rather than a methodological flaw. The unmet clinical need concerns granularity: with the exception of Muehlematter and colleagues, who reported a per-vertebra area under the curve of 0.64 in 58 patients [31], most existing CT-based systems output a single scalar per patient rather than a level-specific risk for L1 through L5. This is a scope decision in each prior work rather than a methodological flaw, but it leaves the per-vertebra setting under-explored even though the clinical question that motivates surgical or augmentation planning is intrinsically segmental: which of L1 to L5 is most likely to fail next. The first methodological gap concerns cohort type. Performance on routine clinical lumbar spine CT and on opportunistic abdominal CT is rarely reported head to head on matched populations from the same institution, even though the two acquisition contexts differ in voxel size, anatomical field of view and clinical indication. The second methodological gap concerns evaluation honesty. Many imaging machine learning studies suffer from subtle leakage through feature selection, preprocessing or decision-threshold selection performed on the full dataset prior to cross-validation; at sample sizes typical of single-centre vertebral cohorts, even small leakage inflates apparent performance by 0.03 to 0.08 AUC [32].

The present study addresses both methodological gaps and the unmet per-vertebra clinical need. We develop a per-vertebra CT biomarker pipeline that predicts fracture risk for each of L1 to L5 individually, that is evaluated independently on a routine clinical lumbar spine CT cohort and a routine abdominal CT cohort assembled at the same institution and acquired across multiple scanners and acquisition periods, and that enforces fully nested leave-one-patient-out cross-validation so that feature selection, preprocessing, hyperparameter tuning and decision thresholds are recomputed inside each outer fold. We further introduce a Two-Stage decomposition that splits the prediction into a patient-level fragility score (how osteoporotic is this skeleton overall) and a within-patient vertebral outlier score (which vertebra deviates most from its neighbours), and we compare it against ElasticNet logistic regression, a softmax approximation of conditional logistic regression and gradient-boosted ranking baselines. The principal contributions of the present work are as follows. The pipeline produces, to our knowledge, the first published per-vertebra CT model with an AUC above 0.70 on a non-surgical primary-prevention cohort; the closest comparable benchmark reported 0.64, although that study used a different cohort and the two values are therefore reference points rather than a controlled head-to-head comparison. A head-to-head comparison of per-vertebra prediction is reported on matched routine clinical lumbar spine CT and opportunistic abdominal CT cohorts from the same institution, with patient-level bootstrap confidence intervals for every reported metric. A systematic ablation of ten methodological variants and sensitivity analyses is reported, none of which produced a statistically significant improvement, thereby establishing a realistic single-centre ceiling at this sample size. Finally, the work reports a clinically interpretable per-vertebra Hit@3 metric and, uniquely among the models evaluated, an expected calibration error consistent with direct probability communication.

## 2. Materials and Methods

### 2.1. Study Design, Setting and Ethics

This was a single-centre retrospective imaging biomarker study conducted on adult patients who underwent either routine clinical lumbar spine CT or routine abdominal CT at Pauls Stradiņš Clinical University Hospital (PSKUS), Rīga, Latvia. All imaging was native (non-contrast) computed tomography acquired for indications unrelated to osteoporosis screening, and the cohort was assembled across multiple CT scanners and multiple acquisition periods at the contributing centre, reflecting routine clinical heterogeneity rather than a controlled imaging protocol. All images were anonymised at source: DICOM headers were stripped of identifying tags and pixel-level burn-ins were removed before any data left the radiology department, in accordance with Article 9 of the General Data Protection Regulation and institutional data protection policy. Anonymised volumes were stored on institutional secure cloud storage with access-controlled credentials, and no identifiable data crossed the hospital boundary at any stage of the pipeline. The study was approved by the institutional ethics committee; written informed consent was waived for retrospective anonymised imaging review.

### 2.2. Cohorts and Outcome Definition

Two independent cohorts were assembled (Table 1). The lumbar cohort comprised 106 patients who underwent routine clinical lumbar spine CT for clinical indications unrelated to osteoporosis screening (such as spinal pain, post-traumatic evaluation or oncological staging) between 2017 and 2024, yielding 478 evaluable L1 to L5 vertebrae after quality control. The abdominal cohort comprised 126 patients in whom routine abdominal CT was obtained for unrelated clinical indications, yielding 589 evaluable vertebrae. The two cohorts were independent and acquired with distinct protocols, across multiple CT scanners and multiple acquisition periods. They were deliberately kept separate and modelled independently throughout. Dedicated lumbar spine CT and abdominal CT are different acquisition types with different underlying data distributions, differing in clinical indication, anatomical field of view and native in-plane resolution, the last by roughly a factor of two and a half in the median (Table 1). The morphological and texture steps described below rescale or resample to a common physical scale, which removes the nominal difference in voxel size but cannot restore detail that was not resolved at acquisition; the distributions therefore remain distinct after preprocessing. Pooling them, or training on one and testing on the other, would therefore mix two distinct input distributions and would not correspond to how such a model would be deployed: in practice the acquisition type is known at inference time, and a model matched to that acquisition type would be applied. We consequently report two parallel analyses rather than one combined model, and no patient contributes to both cohorts. Both cohorts comprise asymptomatic patients with respect to osteoporosis; inclusion was conditional only on the unrelated clinical indication for which the baseline scan was acquired, and we deliberately do not infer the presence or absence of any underlying systemic condition from inclusion alone. Patients with overt anatomical variation of the lumbosacral junction (true L6 vertebra, lumbarised S1 or sacralised L5) were excluded so that the L1-to-L5 labelling was unambiguous in every retained case.

Ground-truth labels were obtained by two independent readers blinded to the imaging analysis: one board-certified diagnostic radiologist and one diagnostic radiology resident. Both reviewers considered the full radiology report of the follow-up imaging study together with the corresponding follow-up images, and classified each vertebra according to the Genant semi-quantitative grading system [33]: a vertebra was labelled as having sustained an incident fracture (target value 1) if it was graded Genant grade 1 (mild) or higher on follow-up imaging and had not been classified as fractured at baseline, and as intact (target value 0) if no such fracture was recorded during clinical follow-up. Follow-up imaging was not restricted to computed tomography: any subsequent imaging study documented during clinical follow-up (computed tomography, plain radiograph or magnetic resonance imaging) was acceptable for incident-fracture adjudication, provided the vertebra of interest was visible. A pre-existing fracture documented at baseline was excluded from analysis at that level. Individual vertebrae were excluded during quality control where endplate reconstruction artefacts or segmentation failures precluded reliable vertebral body isolation; the corresponding per-cohort counts are reported in Appendix A. The TotalSegmentator masks used downstream were spot-checked by both readers on a random subset to confirm anatomical correctness.

### 2.3. Segmentation

**Figure 1:**
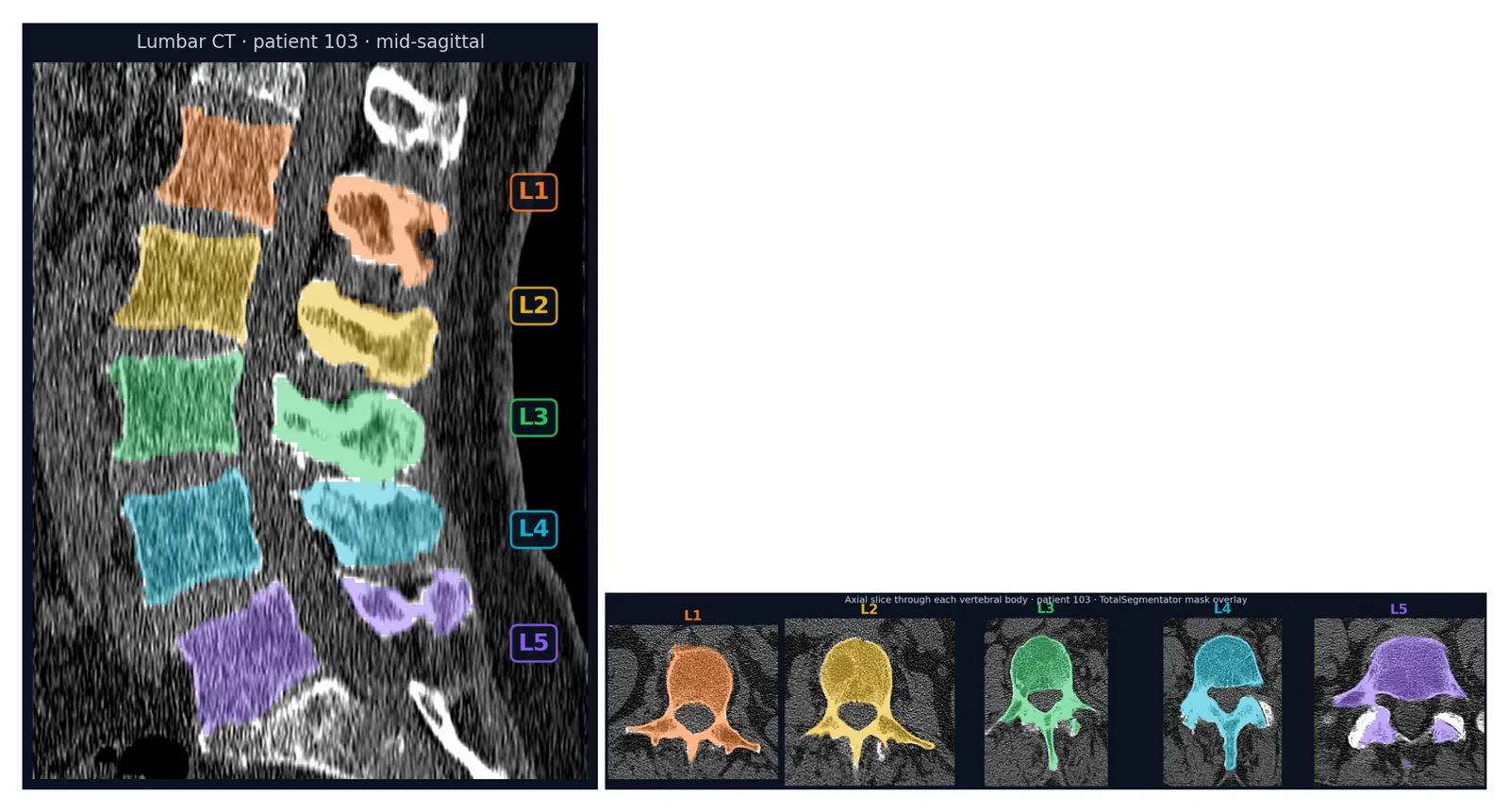
Representative lumbar CT segmentation in a single patient. (**Left**) Mid-sagittal reconstruction with L1 to L5 contours colour coded, one distinct colour per vertebral level. (**Right**) Axial mid-body sections through L1 to L5 at native in-plane resolution. The bony silhouettes of the vertebral body, spinous and transverse processes, pedicles, and intervertebral discs are clearly resolved; the L4 level shows mild trabecular rarefaction visible as a hypo-attenuating central core.

Individual binary masks for L1 through L5 were generated automatically with TotalSegmentator v2 [16], a cascaded three-dimensional nnU-Net [15] trained on 1204 CT volumes covering 104 anatomical structures. The network produces one NIfTI mask per vertebra; no manual editing was performed, and all five lumbar masks were present for every included patient. Representative segmentation is shown in Figure 1.

### 2.4. CT Preprocessing and Region-of-Interest Definition

**Figure 2:**
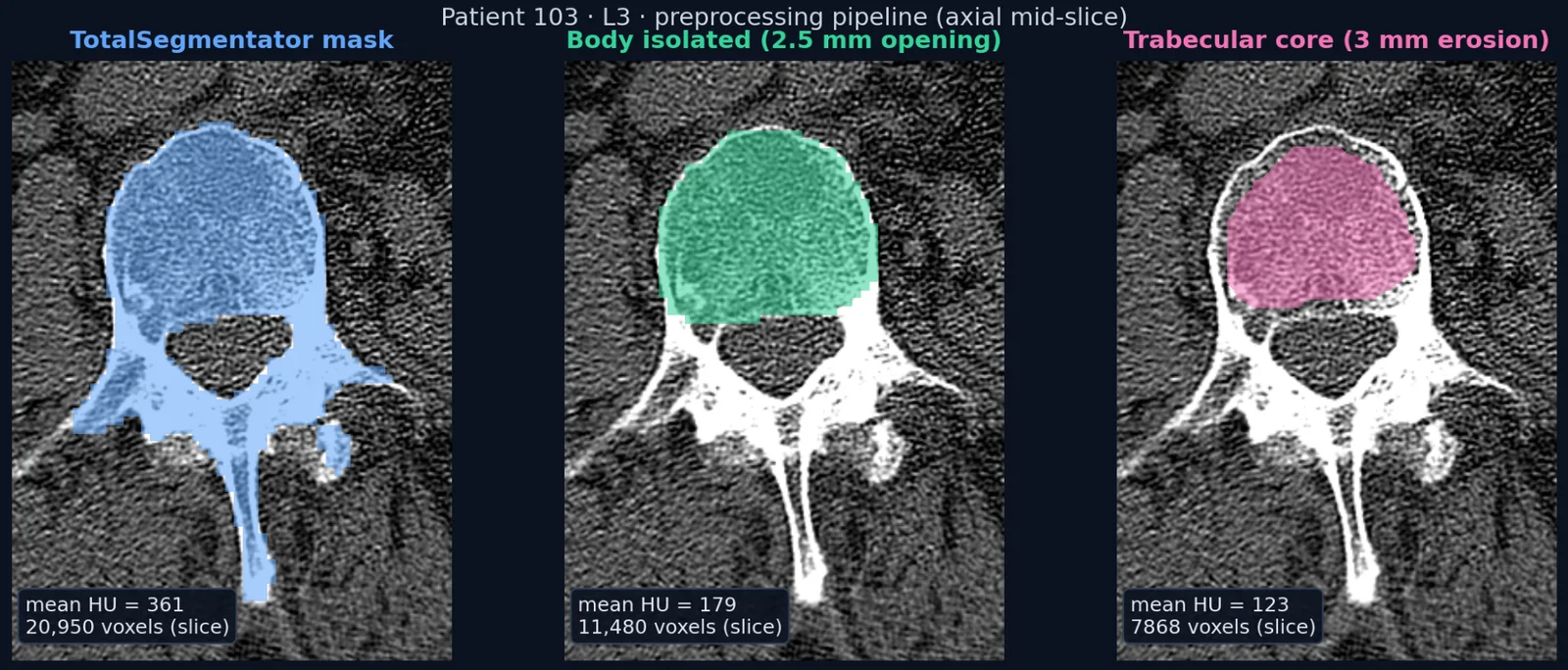
Region-of-interest progression for a single L3 vertebra. (**Left**) Raw TotalSegmentator mask including the vertebral body and posterior elements. (**Centre**) Vertebral body after morphological opening at the 2.5 mm physical scale. (**Right**) Trabecular core after a further 3 mm distance-transform erosion, excluding the cortical shell. Density-based and topological biomarkers are computed on the trabecular core only, while texture features are computed on the full body mask to preserve the spatial extent required by the texture matrices.

Each CT volume was loaded in canonical right-anterior-superior orientation so that axis conventions were deterministic across scans. For every vertebra three nested regions of interest were derived. The first was the raw mask produced directly by the TotalSegmentator. The second was the load-bearing vertebral body, obtained from the raw mask by binary opening with a structuring element scaled to a physical target of 2.5 mm followed by selection of the largest connected component, which removes the spinous and transverse processes while preserving the body. The third was the trabecular core, obtained by an additional 3 mm distance-transform erosion of the body mask to exclude the cortical shell. The opening radius was rescaled to the minimum in-plane voxel spacing for each scan, ensuring consistent morphological scale across acquisitions with native in-plane resolutions ranging from 0.3 to 1.0 mm and through-plane resolutions ranging from 0.5 to 3.0 mm. For texture computation the volumes were additionally resampled to isotropic 1 mm voxel spacing using B-spline interpolation, with a fixed grey-level bin width of 25 Hounsfield units, in compliance with the recommendations of the Image Biomarker Standardisation Initiative [34]. Figure 2 shows the progression from raw segmentation to the trabecular core region of interest.

### 2.5. Quantitative Imaging Biomarkers

A panel of 505 imaging biomarkers per vertebra was extracted, organised in eleven thematic groups (Table 2). Three biomarker families were designed de novo for this study and motivated by biomechanical considerations.

The first novel family is low-density topology. Mean density averages out focal weak zones, so a vertebra with overall mean attenuation of 120 Hounsfield units but a single contiguous cluster below 100 Hounsfield units extending from the superior to the inferior endplate carries a direct fragility pathway for crack propagation under axial load. We therefore enumerated connected components of the sub-threshold core mask at three densities (50, 100 and 150 Hounsfield units) and additionally tested whether any cluster bridged the top and bottom 20% of vertebral height, recording this as a binary endplate-to-endplate bridge indicator.

The second novel family is sub-endplate vulnerability. Vertebral compression fractures preferentially initiate at or near the endplates, where compressive forces concentrate [35]. We therefore extracted separate density and low-attenuation statistics for the superior 15% and inferior 15% axial slabs of the body, rather than averaging across the full vertebral extent, together with the ratio between endplate slab attenuation and core attenuation and the superior-to-inferior asymmetry of these slabs.

The third novel family is radial heterogeneity. Age-related bone loss affects the central trabecular core preferentially while sparing the periphery adjacent to the cortex, so a vertebra may maintain a near-normal mean attenuation while developing critical central rarefaction. We defined three concentric zones from the normalised body distance transform and computed mean attenuation in each zone, together with the outer-to-inner attenuation ratio.

### 2.6. Within-Patient Feature Engineering

Fracture risk is intrinsically a relative quantity. A trabecular attenuation of 100 HU is markedly low for a healthy 35-year-old man but close to average for an 80-year-old woman with established osteoporosis. To expose this structure to the learner without recourse to demographic covariates, ten families of within-patient transforms were applied to every numeric biomarker, computed independently for each patient over their five lumbar levels.

The motivation for within-patient transformations is that fracture risk for a given vertebra depends partly on how that vertebra compares with the patient’s own remaining spine, since age-related and disease-related shifts in mean trabecular attenuation move the entire lumbar column together [10,11,12]. Each biomarker was therefore first re-expressed as a relative z-score against the patient mean and standard deviation, and ranked from one to five within the patient. Binary indicators recorded whether a given vertebra was the patient’s minimum or maximum for the biomarker. Sequential gradients along the spine and the deviation from the average of the two adjacent levels (the local residual) captured abrupt focal transitions. A second difference along the spine (the curvature) detected localised dips in the attenuation profile. Healthy lumbar spines exhibit a smooth, predominantly cranio-caudal density and morphometry gradient documented in quantitative CT cohort studies [9,13]. We therefore additionally computed a per-patient linear-trend residual that removes this expected gradient and isolates vertebrae deviating from their patient’s expected pattern. Distances to the patient minimum, maximum and median were recorded as further within-patient context. For five core biomarkers (mean attenuation, core mean, bone-to-total-volume ratio at 100 HU, connectivity density, and superior-tertile core attenuation) we additionally exposed the values of the immediately previous and next vertebrae and the deviations from each neighbour. Patient-level aggregates (mean and standard deviation across L1 to L5), density–architecture interaction products such as mean attenuation multiplied by largest-component ratio, shape-density coupling such as mean attenuation per unit height, log transforms for highly skewed quantities, and one-hot indicators for vertebral level were also added. The resulting engineered pool comprised 1293 features. All within-patient transforms are by construction safe under leave-one-patient-out cross-validation because they use only information local to a single patient’s five vertebrae.

### 2.7. Cross-Validation Protocol

All evaluations were performed under patient-wise leave-one-patient-out cross-validation. In each outer fold all evaluable vertebrae of one patient were held out, the remaining 105 patients in the lumbar cohort and 125 patients in the abdominal cohort were used for training, and predictions were generated for the held-out vertebrae. This protocol guarantees strict separation between training and test patients and ensures that the model always predicts for a previously unseen patient.

To prevent subtle leakage, every data-dependent decision was made inside the training split of each outer fold. Feature selection used univariate AUC ranking followed by correlation-based pruning at an absolute Pearson coefficient of 0.95, computed on the training patients only. Preprocessing replaced infinite values with missing values, applied percentile clipping at the 0.5th and 99.5th percentiles with bounds fitted on training data and applied identically to validation, performed median imputation, and finally robust-scaled the features. Hyperparameters were tuned by inner five-fold grouped cross-validation on the training patients, and the classification threshold for F1 was selected on inner out-of-fold predictions, never on the held-out patient.

### 2.8. Machine Learning Models

#### 2.8.1. ElasticNet Logistic Regression

A regularised logistic regression with combined L1 and L2 penalty was trained with the saga solver and balanced class weights. The hyperparameter grid spanned regularisation strength values of 0.01, 0.1, 1.0 and 10.0 and L1-to-L2 mixing ratios of 0.1, 0.5 and 0.9, giving twelve combinations evaluated under the inner cross-validation; this conditional grid search over a small number of competing model configurations is a coarse-grained, deterministic instance of the multicriteria conditional optimisation framework formalised in [36]. Four feature-selection variants were tested in parallel: univariate AUC ranking, mutual-information ranking, an averaged AUC-and-mutual-information hybrid, and Boruta wrapper selection.

#### 2.8.2. Softmax-Ranking Approximation of Conditional Logistic Regression

Let $X_{i}\in R^{n_{i}\times p}$ and $y_{i}\in {\left\{0,1\right\}}^{n_{i}}$ denote the feature matrix and label vector for patient *i*, where $n_{i}$ is the number of evaluated vertebrae (typically five). The softmax-ranking model learns coefficients $\beta \in R^{p}$ by maximising

(1) $$L\left(\beta \right)\;=\;\sum_{i}\left[\sum_{j:\;y_{ij}=1}X_{ij}\beta \;-\;k_{i}\;\log\;\sum_{\ell =1}^{n_{i}}\exp\left(X_{i\ell }\beta \right)\right]\;-\;\lambda \;{∥\beta ∥}_{2}^{2},$$

where $k_{i}=\sum_{j}y_{ij}$ is the number of incident fractures in patient *i*. The expression in Equation (1) is an exact conditional likelihood for patients with a single incident fracture and a softmax approximation otherwise; the patient-specific intercept cancels in the conditional, so additive scanner-level effects common to all five vertebrae of a patient do not influence β. Patients without any incident fractures contribute zero gradient and are therefore excluded from likelihood maximisation, but their coefficients are still applied at inference. Optimisation used L-BFGS-B with the analytical gradient, and the regularisation strength λ was tuned by inner two-fold grouped cross-validation over the values $10^{-3}$, $10^{-2}$, $10^{-1}$, 1 and 10.

#### 2.8.3. Two-Stage Model

The Two-Stage model decomposes the prediction into two orthogonal signals. Stage 1, the patient-level fragility score, is a logistic regression trained on patient-aggregated quantities only (the per-patient mean and standard deviation of selected biomarkers and muscle summaries) with a patient-level label set to 1 if any of L1 to L5 sustains a fracture. Its output is a logit score for the systemic fragility of each patient. Stage 2, the vertebral outlier score, is an ElasticNet trained exclusively on within-patient features (relative z-scores, ranks, minimum and maximum indicators, sequential gradients, local residuals, curvatures, trend residuals and the neighbour-context block); these features are zero-centred by construction and therefore measure how each vertebra deviates from its own patient’s pattern. The combiner is a meta-logistic regression:

(2) $${\hat{p}}_{ij}\;=\;\sigma \;\left(\alpha \;s_{ij}^{\left(1\right)}\;+\;\beta \;s_{ij}^{\left(2\right)}\;+\;\gamma \right),$$

in which the stage scores $s_{ij}^{\left(1\right)}$ and $s_{ij}^{\left(2\right)}$ are inner-out-of-fold predictions, never in-sample predictions, so that the combiner does not see calibration leakage from either stage. The decision threshold for binary outputs was selected from inner out-of-fold predictions of the combiner itself. Once the combiner had been fixed, both stages were retrained on the full outer-training split and applied to the held-out patient.

#### 2.8.4. LightGBM Ranker

A gradient-boosted-tree ranker with the LambdaRank objective, treating each patient as a ranking group of five vertebrae, was included as a non-linear ranking baseline. The model was configured with 100 trees, maximum depth 5, learning rate 0.05 and 15 leaves.

### 2.9. Evaluation Metrics

Discrimination was assessed by the area under the receiver-operating-characteristic curve (AUC), the area under the precision-recall curve, F1 at the selected threshold, precision, recall and specificity. Within-patient ranking was assessed by the concordance index, defined as the fraction of correctly ranked fracture-versus-intact pairs within each patient, and by Hit@*K* for K∈{1,2,3}, defined as the fraction of fracture-bearing patients whose *K* highest-scoring vertebrae include at least one actual incident fracture. Probabilistic calibration was quantified by the expected calibration error across ten equal-width probability bins. Every metric was additionally recomputed over 2000 patient-level bootstrap resamples to obtain bias-corrected 95% confidence intervals.

### 2.10. Software and Reproducibility

The pipeline was implemented in Python 3.11 (Python Software Foundation, Wilmington, DE, USA) using scikit-learn 1.3 (INRIA, Le Chesnay, France), PyRadiomics 3.0 (Computational Imaging and Bioinformatics Laboratory, Harvard Medical School, Boston, MA, USA) [37], LightGBM 4.0 (Microsoft Corporation, Redmond, WA, USA), SimpleITK 2.3 (Insight Software Consortium, open-source), BorutaPy 0.4.3 (open-source) and TotalSegmentator v2 (University Hospital Basel, Basel, Switzerland) [16]. All random seeds were fixed for reproducibility.

## 3. Results

### 3.1. Primary Discrimination Performance

**Figure 3:**
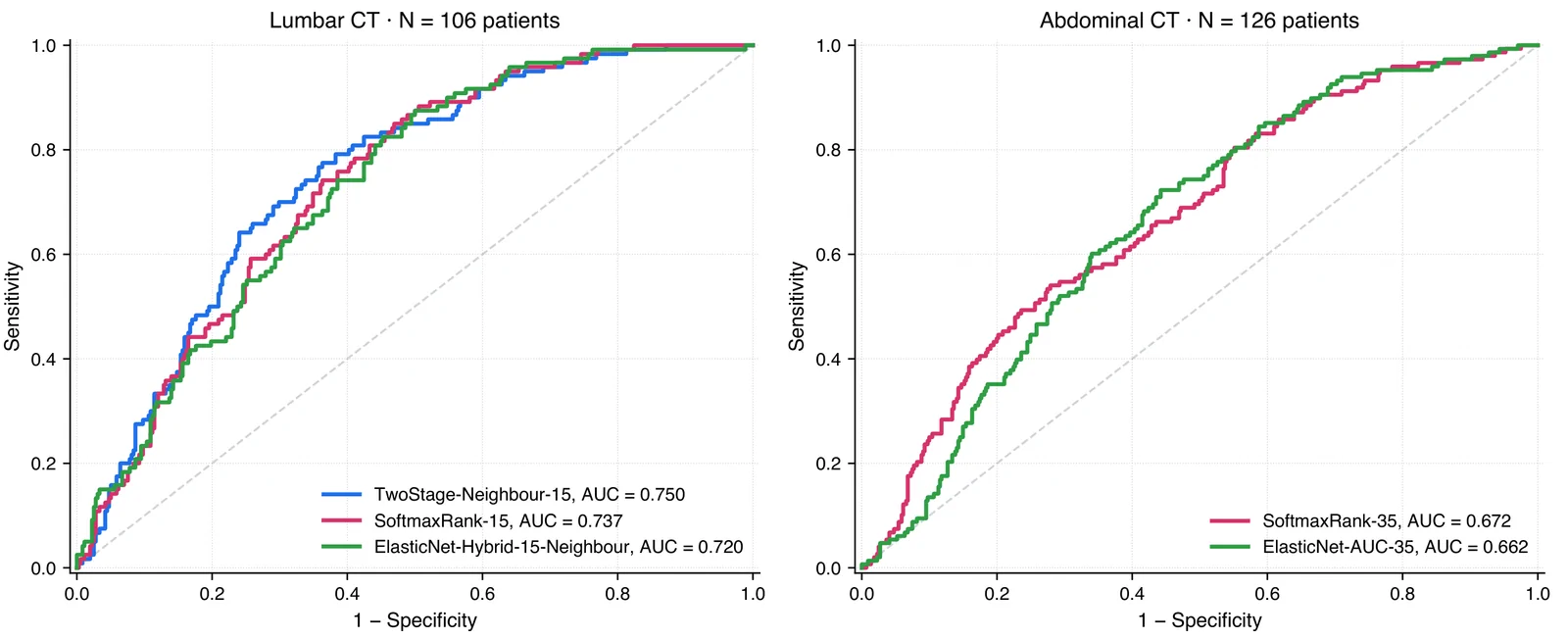
Receiver-operating-characteristic curves for the leading models on each cohort, three on lumbar CT and two on abdominal CT, computed from pooled out-of-fold predictions across all leave-one-patient-out folds. (**Left**) Lumbar CT. (**Right**) Abdominal CT. Each model is identified by colour in the panel legend, where its AUC is also given. The dashed diagonal represents the chance level. The Two-Stage model with neighbour-context features achieves the best discrimination on lumbar CT; on abdominal CT the softmax-ranking model and ElasticNet are closely matched.

**Figure 4:**
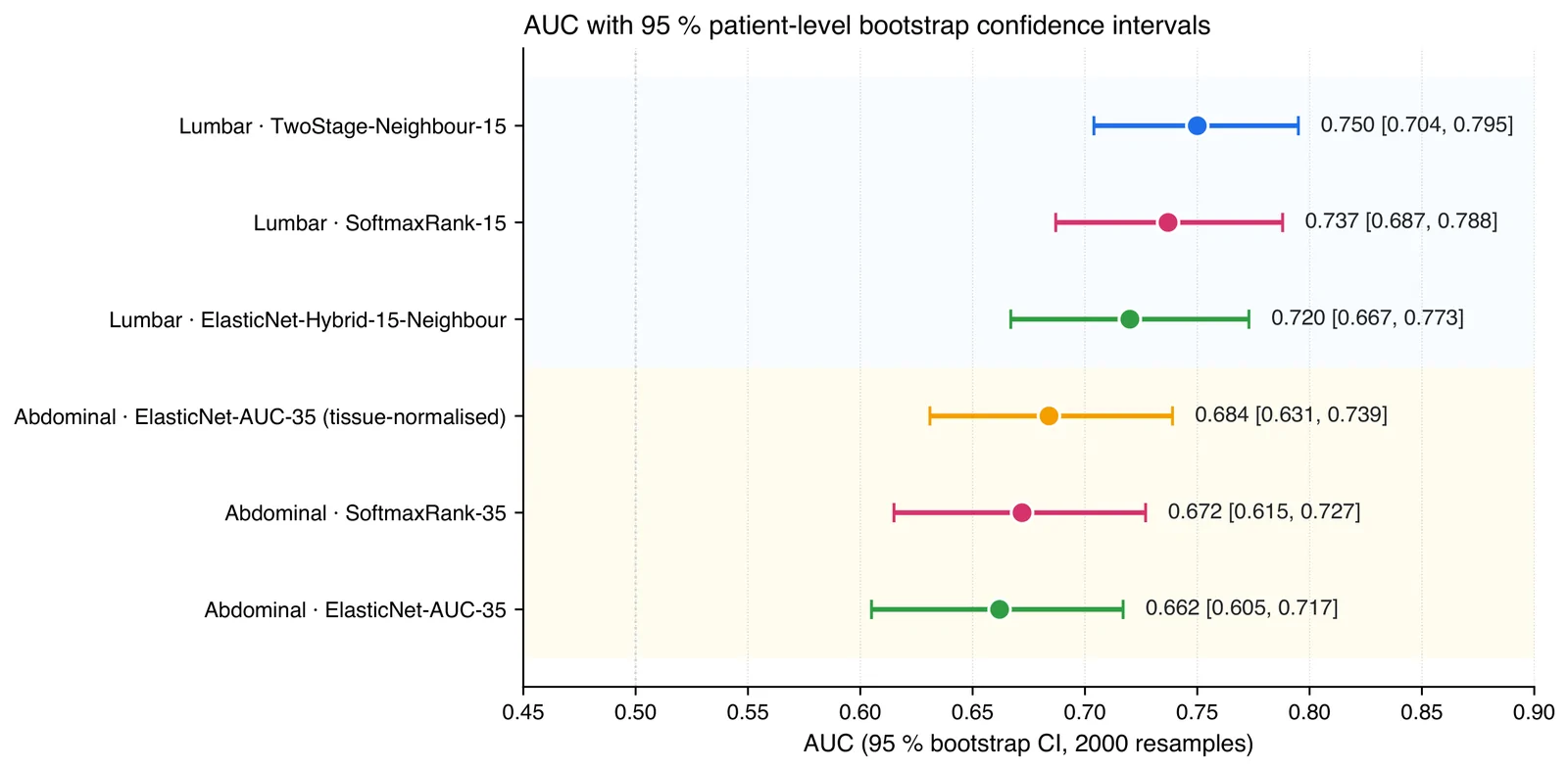
Forest plot of AUC point estimates with 95% patient-level bootstrap confidence intervals (2000 resamples). The pale blue band groups the lumbar-CT models and the pale yellow band the abdominal-CT models. Each marker is the point estimate and the horizontal bar is its confidence interval; numerical values are shown next to each marker. The intervals overlap substantially within each cohort, confirming that the differences between models observed at this sample size are not statistically significant in isolation.

Table 3 summarises the best-performing model on each cohort together with its two strongest competitors, with patient-level bootstrap 95% confidence intervals. On the lumbar cohort the Two-Stage model with neighbour-context features and 15 selected features (designated TwoStage-Neighbour-15 throughout) was numerically best across AUC, F1 and concordance index. Its 95% confidence interval overlapped the softmax-ranking model on every metric, so the superiority observed at this sample size is numerical and consistent rather than statistically significant. On the abdominal cohort the softmax-ranking model with 35 selected features was the strongest single predictor; an internally tissue-normalised ElasticNet variant, in which every Hounsfield-unit-valued feature was divided by the mean of psoas and paraspinal attenuation, gave the best numerical AUC of 0.684 but its confidence interval also overlapped the corresponding baseline. Discrimination is illustrated by receiver-operating-characteristic curves in Figure 3 and by the AUC forest plot in Figure 4.

### 3.2. Sensitivity to the Number of Selected Features

Optimal feature counts differed between cohorts (Table 4). The cleaner lumbar signal was concentrated in a small set, with the optimum at 15 features, whereas the noisier abdominal signal benefited from broader coverage and reached its optimum at 30 to 35 features. For the Two-Stage model on lumbar CT, increasing the feature count from 15 to 20 degraded AUC by 0.016, confirming that Stage 2 outlier detection benefits from a focused rather than a wide feature set.

### 3.3. Stability of the Selected Biomarkers

Feature selection was highly reproducible across the 106 lumbar leave-one-patient-out folds. A stable core of features entered the model in 100% of folds, with only twenty-four unique features ever appearing in the top fifteen across all folds (Table 5). The most consistently selected features were dominated by regional trabecular density (the superior, inferior, anterior and posterior tertile means of the trabecular core, the global mean Hounsfield value and the 25th percentile of the core), by trabecular network architecture (the bone-to-total-volume ratio at 100 HU, the largest-component ratio of the connected trabecular network, and connectivity density), and by two interaction products (mean attenuation multiplied by largest-component ratio, and the fraction of voxels below 100 HU multiplied by connectivity density). Classical texture features were rarely retained on the lumbar cohort. On the noisier abdominal cohort the top thirty-five was more diverse, with 53 unique features entering across folds and a comparably stable core drawn from that wider pool, and included texture and fractal architecture in addition to density.

### 3.4. Probabilistic Calibration

**Figure 5:**
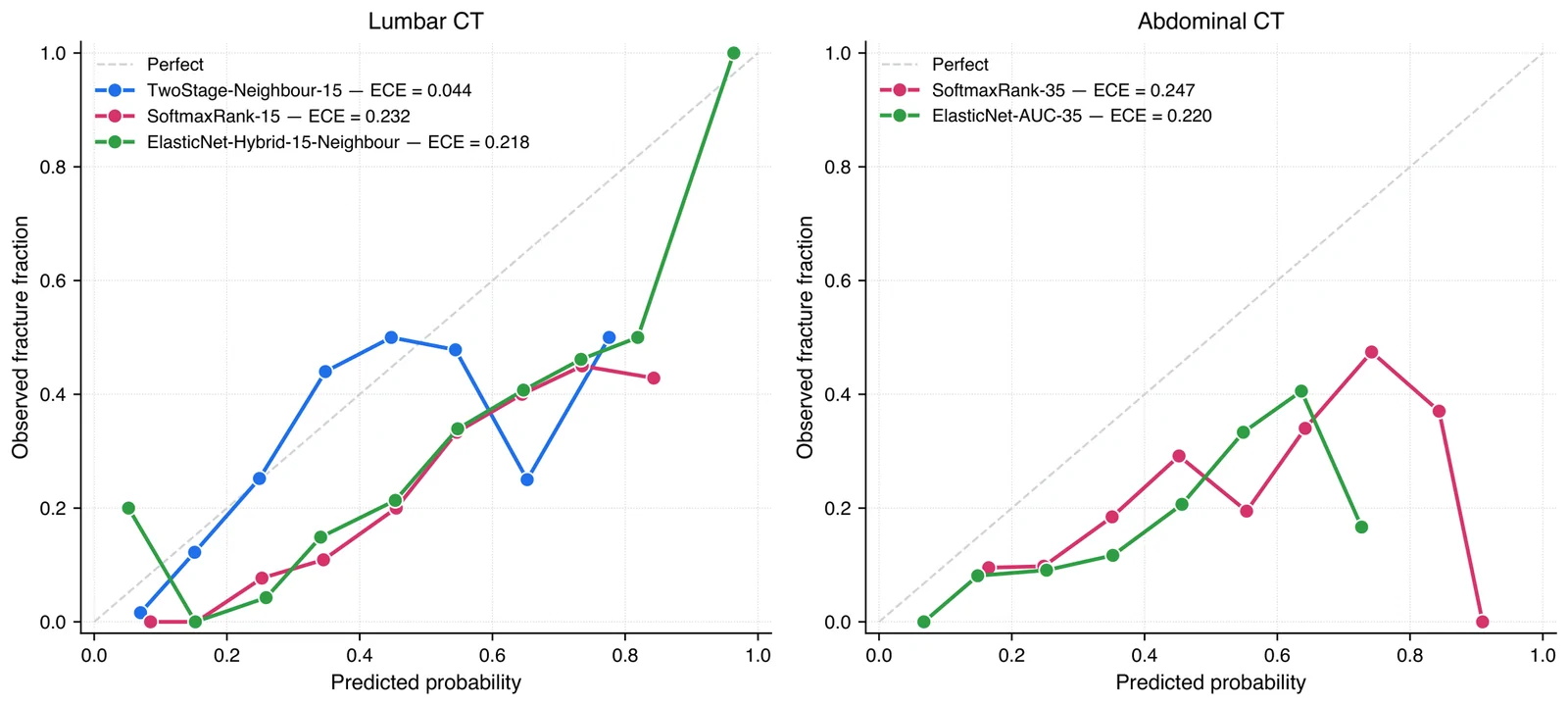
Reliability curves comparing predicted to observed fracture fractions across ten equally spaced probability bins. (**Left**) Lumbar CT. (**Right**) Abdominal CT. The diagonal corresponds to perfect calibration. The Two-Stage model traces the diagonal across the full range on lumbar CT, while the softmax-ranking and ElasticNet models produce probabilities concentrated near the centre of the range and would require explicit recalibration before clinical communication.

The expected calibration error separated the models clearly (Table 6 and Figure 5). Only the Two-Stage model produced well-calibrated probabilities, with an expected calibration error of 0.044 on the lumbar cohort. Outputs from the softmax-ranking model and from ElasticNet clustered in the 0.2 to 0.8 range and would require post hoc Platt or isotonic recalibration before they could be communicated as absolute risk to a clinician.

### 3.5. Within-Patient Ranking and Hit@*K*

**Figure 6:**
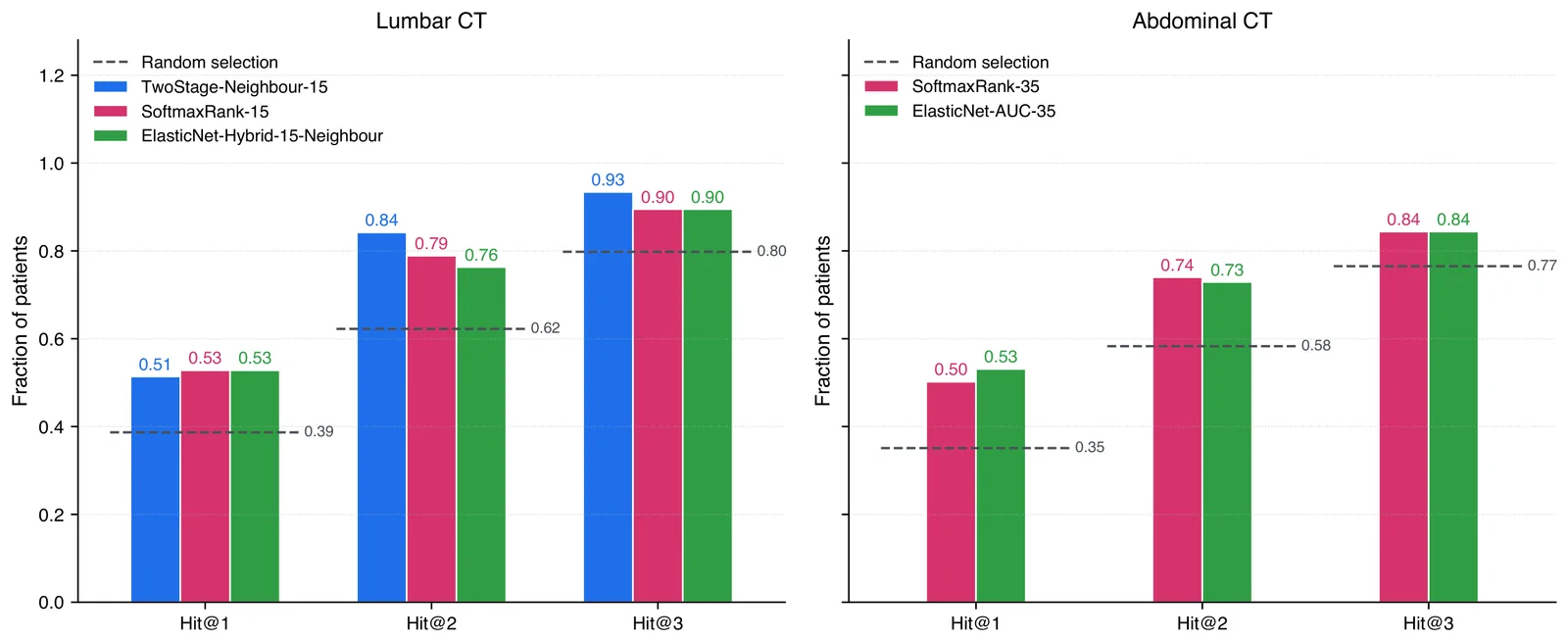
Hit@*K* performance for K∈{1,2,3}, that is the fraction of fracture-bearing patients whose *K* highest-scored vertebrae include at least one actual incident fracture. Dashed horizontal segments mark the expectation under random selection at each *K*, computed patient by patient from the number of evaluable levels and the number of incident fractures of that patient. On lumbar CT the Two-Stage model reaches Hit@3 = 0.93, 13.6 percentage points above the corresponding random expectation of 0.80.

The clinically important question of how often a prophylactic strategy targeting the *K* most-suspect vertebrae would cover an actual fracture was assessed by Hit@*K* (Table 7 and Figure 6). On lumbar CT the Two-Stage model reached a Hit@3 of 0.934; that is, in 93% of fracture-bearing patients at least one vertebra that went on to fracture was among the three highest-risk vertebrae. The expectation under random selection was computed patient by patient, as one minus the hypergeometric probability of drawing no fractured level, because both the number of evaluable levels and the number of incident fractures vary between patients: fracture-bearing patients contribute 4.38 evaluable levels on average, and 30 of the 76 sustained more than one incident fracture. This gives a random Hit@3 of 0.798, so the absolute improvement is 13.6 percentage points. The corresponding random expectations at K=1 and K=2 are 0.387 and 0.622. The model estimate and the random expectation are computed over the same set of 76 fracture-bearing patients. Inspecting the per-patient concordance distribution further shows that 34% of lumbar patients achieved a perfect ranking under the Two-Stage model and only 17% performed worse than chance.

### 3.6. Error Analysis

**Figure 7:**
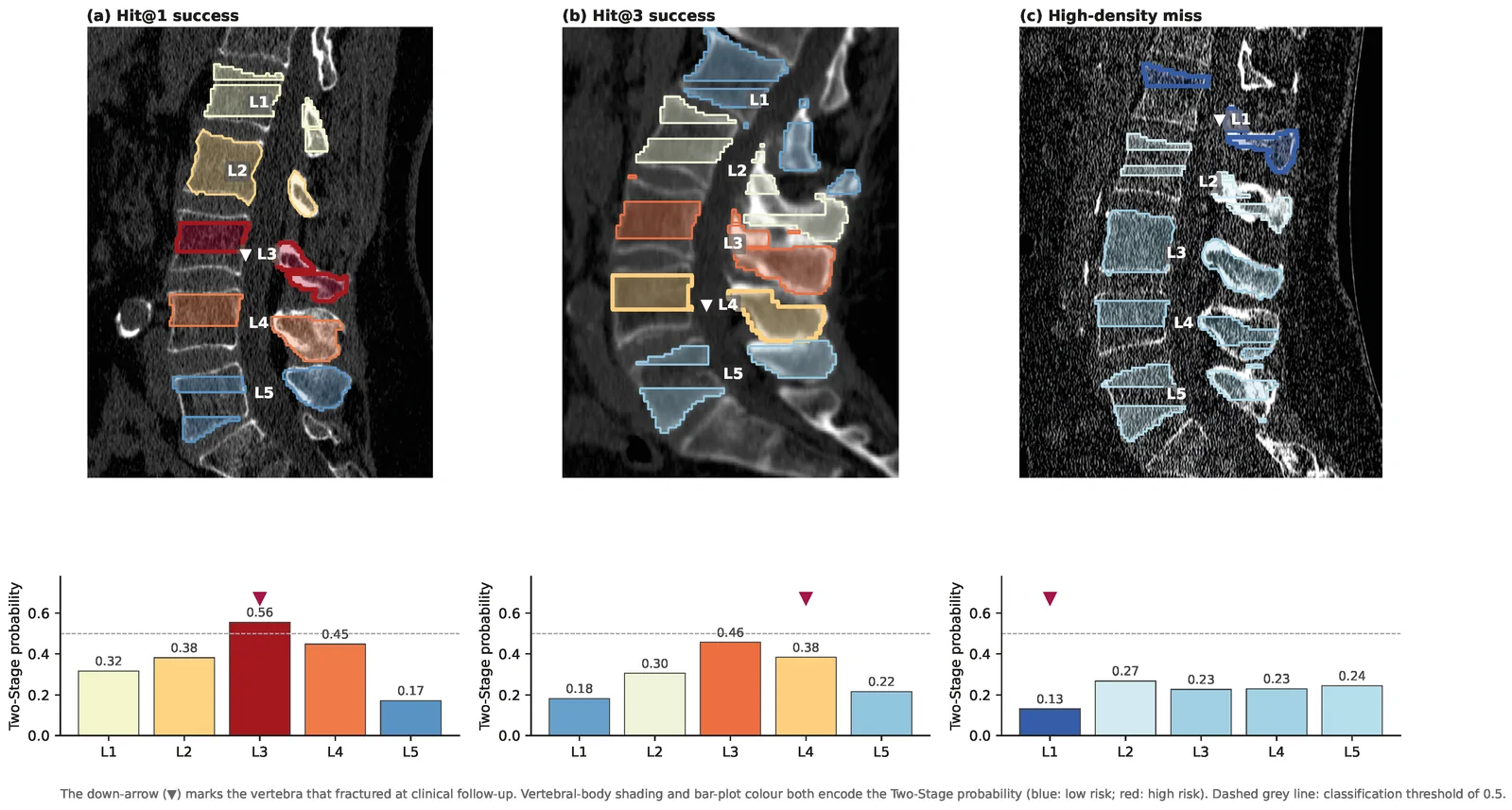
Three representative lumbar-CT cases. Each panel shows the mid-sagittal slice with L1 to L5 contoured in colour by the predicted Two-Stage fracture probability, on a continuous scale in which cooler colours denote lower and warmer colours higher predicted risk, together with a small bar plot of the per-vertebra risk score; the down-arrow marks the vertebra that sustained an incident fracture at clinical follow-up, drawn in white on the computed tomography panels and in red above the corresponding bar. (**a**) Successful Hit@1: a single L3 fracture is correctly ranked first with probability 0.56, well separated from the lowest-risk L5 (0.17). (**b**) Successful Hit@3: the L4 fracture is ranked second of five with probability 0.38; the model places it inside the prophylactic top-three window even though it does not return it as the single most-suspect vertebra. (**c**) Consensus false negative: an L1 fracture occurs in a vertebra with mean attenuation of 253 Hounsfield units, which is well above the osteoporotic range. The model assigns the lowest probability of all five vertebrae, illustrating the failure mode shared by all evaluated models on dense vertebrae and consistent with traumatic or atypical fracture mechanisms outside the intended scope of an osteoporosis-oriented predictor.

Vertebrae that fractured but were missed by every model evaluated in the full model sweep for that cohort, not only by the three leading configurations reported in Table 3 (consensus false negatives), numbered nine on the lumbar cohort and sixteen on the abdominal cohort. In every consensus-false-negative case the baseline vertebra had a mean attenuation of between 200 and 496 Hounsfield units, far above the osteoporotic range. This pattern is consistent with traumatic, pathological or metastatic fracture mechanisms rather than with osteoporotic fragility fractures, that is, with a failure mode outside the intended scope of the model. Consensus false positives were more common on the abdominal cohort (263 vertebrae, 45% of all evaluable vertebrae in that cohort) than on the lumbar cohort, in line with the lower overall discrimination on opportunistic data. Three representative cases that span the clinical regimes encountered in the cohort are illustrated in Figure 7.

### 3.7. Methodological Ablations

To establish a realistic ceiling at this sample size, ten methodological variants and sensitivity analyses were systematically evaluated against the corresponding baseline (Table 8). None produced a statistically significant improvement. The pattern is consistent with the well-documented behaviour of neural-network ensembles and related multi-model architectures on small clinical-imaging cohorts, where the marginal benefit of an additional model component is generally bounded by the cohort’s effective sample size rather than by the architecture itself [38,39]. The strongest numerical gain (an internal tissue normalisation with the mean of psoas and paraspinal attenuation as denominator on the abdominal cohort) raised AUC by 0.022 but its 95% confidence interval overlapped the baseline. Several variants were neutral or marginally negative because the existing engineered pool already captured the corresponding signal: a concordance meta-feature aggregating the within-patient minimum indicators added nothing because the individual indicators outranked their summary; a cortical-trabecular gradient profile added too few candidate features to clear the 15-feature top-list; and additional narrow neighbour features were redundant with the existing local-residual block.

With 106 to 126 patients per cohort and 95% confidence intervals close to ±0.05 AUC, no single methodological tweak can be expected to produce a provable improvement at the present scale. The binding constraint is therefore data volume rather than methodology.

## 4. Discussion

### 4.1. Principal Findings

This study establishes three primary results. First, on routine clinical lumbar spine CT, a Two-Stage decomposition that separates patient-level fragility from within-patient vertebral outlier status achieves a per-vertebra AUC of 0.750 and, uniquely among the tested models, well-calibrated probabilities, with an expected calibration error of 0.044 against 0.232 for the softmax-ranking model and 0.218 for ElasticNet. The probability output can therefore be reported directly as a per-vertebra fracture risk over the available follow-up, without an additional recalibration step. Second, on opportunistic abdominal CT, the Two-Stage model does not transfer, reaching an area under the curve of only 0.632 against 0.672 for the softmax-ranking model, because the patient-level fragility signal is insufficiently clean; in this setting a softmax-ranking model that automatically cancels patient-level scanner effects is preferable, with an AUC of 0.672. The two acquisition contexts therefore call for different modelling strategies, even though both cohorts are drawn from the same institution and the same clinical population. The decision to model the two cohorts separately rests primarily on their documented acquisition differences rather than on this reversal, since with overlapping confidence intervals the ordering of closely matched models should not be over-interpreted; the reversal is nonetheless consistent with the two acquisition types presenting different input distributions. Pooling the cohorts, or transferring a model trained on one to the other, would combine two distinct input distributions and would also misrepresent deployment, in which the acquisition type is known in advance and the matched model would be applied. Intermediate designs exist and are worth stating explicitly: a single model could be fitted with acquisition type as a covariate, or in a stratified or hierarchical form that shares some parameters across acquisitions while allowing others to differ. We did not adopt these here because at the present sample size each additional shared parameter is estimated from fewer effective observations per acquisition type, and because the two-model formulation matches the deployment scenario directly. With the larger multi-centre cohorts discussed below, a hierarchical formulation that borrows strength across acquisition types while preserving their differences becomes an attractive alternative, and we regard it as a natural extension rather than a discarded option. Third, the selected biomarker set converged on a small, highly stable core dominated by regional trabecular density and trabecular network architecture; classical texture features were rarely retained on lumbar CT, suggesting that the discriminative signal for fragility-type fractures is carried primarily by attenuation and connectivity rather than by higher-order texture.

### 4.2. Comparison with the Prior Literature

A small body of prior research has addressed per-vertebra CT prediction in primary, non-surgical patients on cohorts of comparable size; the most directly comparable is the 58-patient texture analysis report of Muehlematter and colleagues, which we treat as one reference point among several rather than as a dominant benchmark given the small cohort. The architectural differences between the present pipeline and that earlier work—explicit modelling of within-patient ranking through a softmax-ranking objective and a dedicated outlier stage, the inclusion of trabecular network architecture and biomechanically motivated low-density topology and sub-endplate vulnerability biomarkers in addition to texture, and a fully nested cross-validation protocol that prevents subtle leakage at small sample sizes—are consistent with the per-vertebra AUC difference observed here, but a head-to-head conclusion on cohorts of this size should be interpreted with appropriate caution.

The present per-vertebra AUC of 0.750 is also competitive with patient-level CT prediction studies on substantially larger cohorts. Pickhardt and colleagues reported a 2-year patient-level AUC of 0.73 from L1 attenuation, L3 muscle attenuation and abdominal fat on 9223 asymptomatic adults [12], and Lee and Pickhardt reported a concordance index of 0.70 from L1 trabecular Hounsfield units alone in 507 patients [10]. Dieckmeyer and colleagues reported an AUC of 0.75 with level-specific volumetric bone mineral density thresholds on 105 patients, an independent cohort of comparable size [20]. Allaire and colleagues achieved an AUC of 0.804 from finite-element-derived vertebral compressive strength in a case-control study of 88 patients [17]. Habitat radiomics with k-means clustering of vertebral sub-regions, recently reported by Zhang and colleagues in 377 patients, produced a concordance index of 0.748 in validation [23]; that figure is a patient-level concordance index and is therefore not directly comparable with our within-patient pairwise value of 0.693 despite a larger cohort. Higher patient-level AUCs near 0.82 to 0.90 have been reported for models trained on much larger cohorts. These include end-to-end deep neural networks, as in Kong and colleagues with 1709 and 4059 patients [18,19]; radiomic nomograms, as in Wang and colleagues with 7906 patients [22]; association analyses of computed-tomography-derived osteosarcopenia, as in Tang and colleagues [24]; and conventional machine learning on chest CT, as in Chen and colleagues with 162 patients in a matched test split [40]. None of these studies, however, returns a per-vertebra prediction.

The contrast between our lumbar AUC of 0.750 and our abdominal AUC of 0.672 is consistent with the long-standing observation that opportunistic CT, while useful for patient-level bone-mineral-density screening [11], loses some fine-grained trabecular signal because of variable acquisition protocols and lower effective spatial sampling along the spine axis. Pickhardt and colleagues reported similar drops when moving from routine clinical lumbar-spine imaging to opportunistic abdominal imaging, and Hummel and colleagues recently reported that quantitative CT trabecular texture and paraspinal muscle features added little to bone mineral density alone in women in the AGES cohort [21], in line with the present finding that the marginal value of secondary biomarkers is modest at single-centre sample sizes.

### 4.3. Clinical Relevance

The principal near-term benefit of per-vertebra prediction is opportunistic. The model operates on computed tomography acquired for an unrelated clinical indication, so elevated fracture risk can be flagged without additional imaging, additional radiation dose, additional cost or a dedicated dual-energy X-ray absorptiometry study. In the present cohorts the baseline scan was in every case acquired for a non-osteoporosis indication and the incident fracture was documented later on clinical follow-up imaging; flagging elevated risk on that initial scan would have opened a window for established preventive management before the fracture occurred. We regard this, rather than any interventional application, as the primary clinical rationale for the present work, and it allows patients at risk to enter existing management pathways earlier.

Osteoporosis is managed at the patient level, and pharmacological therapy reduces systemic skeletal fragility rather than protecting an individual vertebra. Level-specific risk mapping therefore does not replace patient-level risk assessment; it adds a segmental dimension to it. Where a vertebra is nevertheless treated mechanically, the level is what defines the intervention, since cement-augmented stabilisation is planned and performed on individual levels [41]. Vertebroplasty and balloon kyphoplasty are established treatments for existing symptomatic osteoporotic fractures: radiographic cement leakage is relatively common but asymptomatic in the majority of cases, and serious complications such as symptomatic pulmonary cement embolism, neurological injury or adjacent-level fracture are rare [42,43], with balloon kyphoplasty carrying a lower leakage and adverse-event risk than vertebroplasty [44]. Their prophylactic use on a vertebra that has not yet fractured is, by contrast, not established standard care and carries the same procedure-related risks without an equally documented benefit. We therefore present prophylactic level-targeting as a potential future application to be evaluated prospectively, not as a currently recommended pathway.

Within that framing, the Hit@3 of 0.934 obtained on lumbar CT quantifies how well a segmental strategy could be targeted if one were adopted. Interventions of this type are typically considered for one to three levels per patient, and the model’s per-patient ranking would cover at least one future fracture level in 93% of fracture-bearing patients if the three highest-scoring vertebrae were selected, against the 79.8% expected from random selection, computed patient by patient from each patient’s number of evaluable levels and incident fractures. This 13.6-percentage-point improvement, combined with calibrated probability outputs that allow direct risk communication, is the kind of effect that would warrant a dedicated prospective evaluation, including a formal assessment of costs and benefits, which the present study does not attempt. To our knowledge, this is the first study to report per-vertebra Hit@*K* performance for incident osteoporotic fracture prediction; the metric itself is meaningful only at the segmental level and therefore could not be evaluated in the patient-level studies reviewed above.

### 4.4. Why a Two-Stage Decomposition Helps on Dedicated Lumbar CT

The Two-Stage model is motivated by a simple but under-exploited observation: per-vertebra fracture risk is the product of a systemic factor (how osteoporotic the skeleton is overall) and a local factor (which vertebra within that skeleton deviates most from its neighbours). A single flat classifier must compress these two orthogonal signals into one score, and under regularisation it tends to distribute weight diffusely across correlated feature families. Explicit decomposition allows Stage 1 to concentrate on systemic markers (mean density and variability across the lumbar spine, and muscle quality summaries) and Stage 2 to concentrate on within-patient contrasts (relative z-scores, ranks, minimum indicators, and neighbour differences). The neighbour-context features, that is the values of the immediately previous and next vertebrae, are particularly informative for Stage 2 (with an AUC gain of 0.025) but harm flat classifiers because they introduce missing values at the L1 and L5 edges and dilute the global feature pool when no decomposition is applied.

The practical payoff of the decomposition is calibration rather than raw discrimination. Because Stage 1 estimates the systemic component of risk explicitly, the model can express how fragile a given skeleton is in absolute terms, and Stage 2 then places the individual vertebrae within that patient on a common scale. A within-patient ranking model cannot do this by construction: the softmax normalisation removes precisely the shared, patient-level component that carries the absolute level of risk, which is why it orders vertebrae well but yields poorly calibrated probabilities (expected calibration error 0.232 against 0.044 for the Two-Stage model). For a segmental decision, which is the clinical question that motivates per-vertebra prediction at all, an ordering alone is insufficient, since it indicates which vertebra is most suspect but not whether any of them is at appreciable risk. This is the sense in which the Two-Stage model is doing work that its discrimination numbers alone do not display.

### 4.5. Why Softmax Ranking Suffices on Opportunistic Abdominal CT

The abdominal CT result is at first counter-intuitive: a ranking model out-performs a calibrated classifier. The explanation lies in the denominator of Equation (1). Any additive component of a vertebral score that is common to all five vertebrae of a patient, including scanner-dependent Hounsfield unit bias, convolution-kernel-dependent density shift and patient-level body habitus effects, cancels identically in the softmax normalisation. The softmax-ranking model therefore enjoys a form of automatic within-patient normalisation. The same explanation accounts for the parallel observation that internal normalisation by muscle attenuation helps ElasticNet by 0.022 AUC but the softmax-ranking model only marginally: the ranking model has already neutralised the same nuisance signal internally. For abdominal CT, where scanner heterogeneity and acquisition variability are larger than in routine clinical spine imaging, this property is disproportionately valuable.

### 4.6. Methodological Rigour

A distinctive feature of this study is the systematic ablation of ten methodological variants and sensitivity analyses, none of which produced a statistically significant gain. We regard this as one of the most informative parts of the paper. It indicates that within the present single-centre data envelope, the binding constraint is sample size rather than feature engineering or model architecture. The result is consistent with the broader literature: at quantitatively comparable sample sizes, additional bone architecture or paraspinal muscle biomarkers add little or nothing to bone mineral density [21], and feature parsimony rather than feature richness drives generalisable performance. We therefore caution against the common pattern of stacking ever more handcrafted biomarkers in single-centre cohorts.

### 4.7. Limitations

Several limitations qualify the conclusions. The sample of 106 to 126 patients per cohort gives bootstrap confidence intervals approximately ±0.05 AUC wide, so claims of superiority between closely matched models cannot be made with statistical significance at this scale. We therefore make no claim of statistical superiority for the Two-Stage model over the softmax-ranking baseline: the advantage observed here is numerical and consistent across metrics, but the confidence intervals overlap, and the study is powered to characterise the pipeline rather than to rank architectures against one another. This is a statement about discrimination, and it should not be read as implying that the decomposition is redundant. The case for the Two-Stage architecture rests on a property that discrimination metrics do not capture and that the present data do establish: it is the only model in the comparison that returns calibrated absolute probabilities, with an expected calibration error of 0.044 against 0.232 for the softmax-ranking model and 0.218 for ElasticNet. A within-patient ranking model cannot supply this by construction, because the softmax normalisation deliberately cancels the component of risk that is shared by all vertebrae of a patient, which is precisely the component that determines whether a given skeleton is fragile in absolute terms. The decomposition recovers that component explicitly in Stage 1 while retaining within-patient contrast in Stage 2, and it is what allows a per-vertebra output to be reported as an absolute risk rather than only as an ordering. To indicate the scale of this limitation rather than merely assert it, we performed a precision calculation of the conventional kind used to size a future study from the variance observed in a pilot cohort. No additional patients were involved: the calculation is a patient-clustered bootstrap in which the present 106 lumbar patients are drawn with replacement to form simulated evaluation sets of increasing size, so a bootstrap sample of 500 draws still contains at most 106 distinct individuals. On that basis the half-width of the patient-clustered bootstrap interval scales with the inverse square root of cohort size, from ±0.046 AUC at the present cohort size to approximately ±0.02 AUC at a bootstrap size of 500. The scope of this calculation is deliberately narrow, and its reliance on bootstrap resampling of a single small cohort is itself a limitation. It describes the precision attainable in the evaluation and does not predict that discrimination itself would improve with additional training data. Because every patient in a bootstrap sample is a copy of one of the 106 observed patients, the calculation assumes that between-patient variability remains exactly as observed in this single centre; a genuinely multi-centre cohort of 500 real patients would add scanner, protocol and population heterogeneity that resampling cannot reproduce, so 500 is a lower bound on the requirement rather than a sufficient target. We further refrain from projecting the cohort size at which the difference between the two leading models would become statistically significant, because any such projection must assume a true effect size, and the difference observed here has a confidence interval that includes zero. What the calculation does establish is that no realistic single-centre expansion would materially narrow these intervals, which is why multi-centre pooling rather than continued local recruitment is the necessary next step.

All scans originate from a single institution, a single picture-archiving-and-communication -system pipeline and a bounded range of CT scanners. External multi-centre validation is essential before clinical deployment; it could not, however, be completed within the timescale of the present analysis, because each additional site requires separate ethics approval, data-transfer agreements and General Data Protection Regulation compliant anonymisation, including cross-border data-sharing clearance. We therefore present this work as a transparent single-centre benchmark with its statistical limits reported explicitly.

Ground-truth labels were extracted from clinical follow-up imaging rather than from prospectively standardised semi-quantitative morphometry, so label noise is non-zero. Incident fractures were adjudicated on heterogeneous follow-up studies (computed tomography, radiography or magnetic resonance imaging) obtained at clinically determined rather than protocol-fixed times, and the outcome was modelled as a binary indicator, so the fracture-free interval is discarded and all future fractures are weighted equally regardless of when they occurred. Variable follow-up duration therefore introduces label noise whose direction is not fixed: a vertebra labelled intact but observed over only a short interval may fracture subsequently, which attenuates apparent discrimination, whereas imaging prompted by symptoms may preferentially capture events and act in the opposite direction. A time-to-event formulation that exploits the interval information the binary outcome discards is a planned extension. Patient-level dual-energy X-ray absorptiometry T-scores were available for approximately 40% of patients, but deliberately excluded in order to keep the study purely imaging-based, and a future hybrid model that combines DXA with per-vertebra CT is a natural extension. Consensus-false-negative analysis showed that all such failures occur in vertebrae with mean attenuation above 200 Hounsfield units, suggesting traumatic or pathological fracture mechanisms outside the intended scope of an osteoporosis-oriented model. Finally, only the Two-Stage model produced well-calibrated probabilities; the softmax-ranking and ElasticNet models used for abdominal CT would require Platt or isotonic recalibration before direct clinical communication of absolute risk.

### 4.8. Future Work

Four concrete directions follow from the present ceiling. First, multi-centre recruitment should expand each acquisition-specific cohort to at least 500 patients. This target is the size at which the bootstrap-based precision calculation reported above places the confidence intervals for the lumbar cohort near ±0.02 AUC; because that calculation bootstraps the present 106 patients rather than observing new ones, 500 real patients recruited across several centres would be expected to give somewhat wider intervals, and the figure should be read as a minimum rather than a sufficient target. Pooling is understood here as pooling across centres within an acquisition type, not across the two acquisition types, which for the reasons given in the Materials and Methods are modelled separately. This requires the site-by-site ethics, data-transfer and data-protection groundwork described above. Second, the Two-Stage framework is model-agnostic at both stages, so that the linear classifiers could in principle be replaced by gradient-boosted or neural-network alternatives, and the binary outcome could be extended to a time-to-event Cox combiner that exploits fracture-free interval information that the present binary outcome discards. Third, the consensus-false-negative analysis suggests that a preliminary attenuation-based screening filter, which would defer or de-prioritise vertebrae with baseline attenuation well above the osteoporotic range, could sharpen clinical triage by restricting the model to the failure mode it is designed for. We deliberately do not implement such a threshold here: the high-attenuation subgroup in the present cohorts is too small to establish a cut-off that we could validate, and proposing an unvalidated threshold would run counter to the evaluation discipline adopted throughout this work. Fourth, prospective evaluation of whether a level-specific risk output changes management, for example by directing targeted follow-up imaging, would be the most informative confirmation of the clinical utility suggested by the present numbers. Beyond the immediate clinical context, the same opportunistic CT per-vertebra pipeline is directly applicable to younger adults and to war-affected populations in whom routine non-spine CT is acquired for trauma, oncological or general clinical indications and in whom systematic osteoporosis screening is not in place; extending the present methodology to these populations is the explicit motivation of the wider Latvian–Ukrainian bilateral programme under which this study was conducted, and is the planned focus of subsequent work in that programme.

## 5. Conclusions

A fully reproducible per-vertebra fracture prediction pipeline was developed and evaluated on two independent single-centre CT cohorts under fully nested leave-one-patient-out cross-validation. On routine clinical lumbar spine CT, a Two-Stage model that combines a patient-level fragility score with a within-patient vertebral outlier score achieves an AUC of 0.750 and a Hit@3 of 0.934, while producing well-calibrated probabilities, which makes per-vertebra risk communication feasible without further recalibration. On opportunistic abdominal CT, a softmax approximation of conditional logistic regression is the preferred model, with an AUC of 0.672, benefiting from automatic cancellation of patient-level scanner side effects. The selected biomarker set converged on a small, highly stable core of regional trabecular density and trabecular network architecture descriptors.

These results should be read as a single-centre benchmark rather than as a demonstration of architectural superiority. The confidence intervals of the leading models overlap on every metric, so the discrimination advantage of the Two-Stage decomposition is numerical and consistent but not statistically established at this sample size, and the same caution applies to comparisons with prior per-vertebra predictors evaluated on different cohorts. The rationale for the decomposition does not rest on that comparison. It is the only model evaluated here that returns calibrated absolute probabilities rather than an ordering alone, a capability a within-patient ranking model cannot provide by construction, and it is this that makes a per-vertebra estimate reportable as risk. Identifying the individual vertebra most likely to fail is in turn a prerequisite for any level-targeted preventive strategy. Ten candidate methodological variants and sensitivity analyses, including rank fusion, internal tissue normalisation and additional biomechanically motivated biomarkers, produced no statistically significant gain, which locates the binding constraint in data volume rather than in model configuration; a bootstrap-based precision calculation, in which the present patients were drawn with replacement to simulate larger evaluation sets, indicates that of the order of 500 patients would be required to halve the width of the reported confidence intervals. The clearest near-term value of the pipeline is therefore opportunistic: identifying elevated per-vertebra fracture risk on computed tomography already acquired for unrelated indications, without additional radiation, cost or a dedicated densitometric study. Prophylactic level-targeting remains a potential rather than an established application. External multi-centre validation is the necessary next step before any clinical deployment.

## Figures and Tables

**Table 1:** Cohort characteristics after quality control. Pre-existing fractures visible on the baseline CT were excluded from the analysis because the outcome of interest is incident, that is future, fracture.

Cohort	Patients	Evaluable L1–L5 Vertebrae	Will-Fracture/Intact	Median In-Plane Spacing (mm)	Median Slice Thickness (mm)
Abdominal CT (opportunistic)	126	589	148/441	0.78	1.25
Lumbar CT (routine clinical)	106	478	120/358	0.31	1.25

**Table 2:** Principal quantitative imaging biomarker categories; counts are approximate and the listed categories are not an exhaustive partition of the 505 extracted features. Hounsfield units are abbreviated HU. Texture matrices follow the Image Biomarker Standardisation Initiative naming.

Category	Count	Description
Trabecular density	∼60	Mean attenuation, 10th to 90th percentiles, core statistics (mean, standard deviation, skewness, and kurtosis), cortical-shell density
Spatial density profiles	∼20	Anterior, middle, posterior, superior, inferior, left and right regional attenuation
Vertebral morphometry	∼10	Anterior, middle and posterior body heights; wedge index; biconcavity index; total volume
Hounsfield unit fractions	∼8	Fraction of voxels below 50, 100, 150 and 200 HU
Classical texture	∼120	First-order, shape, grey-level co-occurrence, run-length, size-zone, dependence and neighbourhood difference matrices, with Laplacian-of-Gaussian filtered variants at σ=2.0 and 3.0 mm
Trabecular network architecture	∼20	Bone-to-total-volume ratio at 100, 150 and 200 HU; trabecular thickness and spacing; trabecular number; connectivity density; Euler number; fractal dimension; lacunarity; structural anisotropy; surface-to-volume ratio; porosity gradient
Low-density topology	12	Cluster count, largest-cluster fraction, total low-density fraction at three thresholds; endplate-to-endplate bridge indicator
Sub-endplate vulnerability	8	Density and low-attenuation fraction in the superior and inferior 15% slabs; endplate-to-core ratios; superior-to-inferior asymmetry
Radial heterogeneity	4	Inner, middle, outer concentric-zone mean attenuation; outer-to-inner ratio
Muscle and bone–muscle interaction	39	Psoas and paraspinal muscle attenuation, fat fraction below 30 HU, cross-sectional area, volume, left–right asymmetry, and muscle-to-bone density ratios
Quality control and spacing	4	Voxel spacing along three axes and direction cosines for orientation verification

**Table 3:** Primary results under fully nested leave-one-patient-out cross-validation with patient-level bootstrap 95% confidence intervals (2000 resamples).

Cohort and Model	AUC [95% CI]	F1 [95% CI]	Hit@1 [95% CI]	C-Index [95% CI]
*Lumbar CT (106 patients, 478 vertebrae)*				
TwoStage-Neighbour-15	**0.750 [0.704, 0.795]**	**0.549 [0.487, 0.608]**	0.514 [0.426, 0.596]	**0.693 [0.643, 0.743]**
SoftmaxRank-15	0.737 [0.687, 0.788]	0.500 [0.435, 0.562]	**0.528 [0.442, 0.614]**	0.644 [0.585, 0.703]
ElasticNet-Hybrid-15-Neighbour	0.720 [0.667, 0.773]	0.493 [0.429, 0.555]	**0.528 [0.444, 0.612]**	0.644 [0.583, 0.702]
*Abdominal CT (126 patients, 589 vertebrae)*				
SoftmaxRank-35	0.672 [0.615, 0.727]	**0.464 [0.408, 0.519]**	0.502 [0.426, 0.581]	0.620 [0.561, 0.679]
ElasticNet-AUC-35	0.662 [0.605, 0.717]	0.445 [0.390, 0.501]	**0.531 [0.459, 0.610]**	**0.628 [0.571, 0.688]**
ElasticNet-AUC-35 (tissue-normalised)	**0.684 [0.631, 0.739]**	0.452 [0.398, 0.506]	0.515 [0.440, 0.593]	0.622 [0.568, 0.678]

Numerals after model names denote the number of features retained after correlation pruning. “Neighbour” indicates inclusion of the neighbour-context block. “Tissue-normalised” indicates internal normalisation by the mean of psoas and paraspinal attenuation. Bold indicates the best value in each column within each cohort.

**Table 4:** ElasticNet AUC against the number of selected features. Lumbar CT prefers small feature pools; abdominal CT prefers larger ones. Bold indicates the highest AUC in each cohort.

Number of Features	Lumbar AUC (Hybrid)	Lumbar AUC (Univariate)	Abdominal AUC (Univariate)	Abdominal AUC (Mutual Information)
15	**0.737**	0.733	0.643	0.623
20	0.728	0.727	0.653	0.635
25	0.717	0.715	0.653	0.637
30	0.724	0.714	0.656	0.631
35	0.724	0.712	**0.662**	0.621

**Table 5:** Most frequently selected biomarkers across the lumbar leave-one-patient-out folds for the SoftmaxRank-15 model.

Biomarker	Selection Frequency	Category
Superior tertile core mean attenuation	100%	Regional trabecular density
Bone-to-total-volume ratio at 100 HU	100%	Trabecular network architecture
Inferior zone core attenuation	100%	Regional trabecular density
Posterior-tertile core mean attenuation	100%	Regional trabecular density
Global mean attenuation	100%	Trabecular density
Core 25th-percentile attenuation	100%	Trabecular density (low-end)
Anterior tertile core mean attenuation	100%	Regional trabecular density
Posterior zone density	97%	Regional trabecular density
Largest component ratio	97%	Trabecular network connectivity
Connectivity density	91%	Trabecular network architecture

**Table 6:** Expected calibration error in ten probability bins. Bold indicates the lowest calibration error.

Model	Expected Calibration Error
TwoStage-Neighbour-15 (lumbar)	**0.044**
SoftmaxRank-15 (lumbar)	0.232
ElasticNet-Hybrid-15-Neighbour (lumbar)	0.218
SoftmaxRank-35 (abdominal)	0.247
ElasticNet-AUC-35 (abdominal)	0.220

**Table 7:** Hit@*K* and within-patient concordance index. Bold indicates the best value in each column within each cohort.

Model	Hit@1	Hit@2	Hit@3	C-Index
*Lumbar CT*				
TwoStage-Neighbour-15	0.514	**0.842**	**0.934**	**0.693**
SoftmaxRank-15	**0.528**	0.789	0.895	0.644
ElasticNet-Hybrid-15-Neighbour	**0.528**	0.763	0.895	0.644
*Abdominal CT*				
SoftmaxRank-35	0.502	**0.740**	**0.844**	0.620
ElasticNet-AUC-35	**0.531**	0.729	**0.844**	**0.628**

**Table 8:** Ten methodological variants and sensitivity analyses evaluated against the baseline. Each ΔAUC is relative to the corresponding baseline within the same row group.

Extension	Δ AUC	Outcome
Excluding one patient with reconstruction artefacts identified by the audit	−0.028 (baseline shift)	Assessed; not applied in the primary analysis
Replacing Stage 2 ElasticNet with softmax ranking inside the Two-Stage model	−0.014	Rejected (mis-calibrated combiner input)
Merging the historical and new abdominal feature extractions (1644 engineered features)	−0.009 to −0.025	Rejected (different regions of interest, identical names)
Adding ratio-to-neighbour-mean and neighbour z-score features	−0.014 to +0.001	Rejected (redundant with the local-residual block)
Re-selecting features inside inner cross-validation for the softmax-ranking model	−0.002	Neutral (pipeline already nested)
Adding a concordance meta-feature aggregating within-patient minima	+0.0000	Rejected (dominated by component features)
Internal tissue normalisation of all density features by the mean of psoas and paraspinal attenuation (best variant)	+0.022 (CIs overlap)	Numerical only, not significant
Cortical-trabecular gradient profile across five depth bands	+0.0000	Rejected (best gradient feature ranked 61 of 1329)
Adding 34 new endplate, low-density and patient-aggregate features	−0.005 to −0.031	Rejected (diluted the selection pool)
Rank fusion across three diverse models	−0.008	Rejected (component models too correlated)

## Data Availability

The code implementing feature extraction, feature engineering, leave-one-patient-out cross-validation and all models reported in this paper is available from the corresponding author on reasonable request. Raw CT volumes cannot be shared due to data protection restrictions; they are stored on institutional secure cloud storage with access-controlled credentials. Derived tabular feature tables, without any identifiable information, can be shared under a data-use agreement.

## Abbreviations

The following abbreviations are used in this manuscript:

AUC	Area under the receiver-operating-characteristic curve
BV/TV	Bone volume to total volume ratio
CI	Confidence interval
CT	Computed tomography
DXA	Dual-energy X-ray absorptiometry
ECE	Expected calibration error
FRAX	Fracture Risk Assessment Tool
GDPR	General Data Protection Regulation
HU	Hounsfield units
IBSI	Image Biomarker Standardisation Initiative
LOPO	Leave-one-patient-out cross-validation
PACS	Picture archiving and communication system
PR-AUC	Area under the precision–recall curve
PSKUS	Pauls Stradiņš Clinical University Hospital
ROI	Region of interest
RSU	Rīgas Stradiņa Universitāte

## Appendix A

### Appendix A.1. Outline of the Nested Cross-Validation Protocol with the Two-Stage Model

For each outer leave-one-patient-out fold, the patient $i^{*}$ to be predicted is left out and the remaining patients form the outer training set. Feature selection (univariate AUC ranking followed by correlation pruning at an absolute Pearson coefficient of 0.95) is computed exclusively on the outer training set. Inner out-of-fold scores for Stage 1 (patient-level fragility) and Stage 2 (within-patient vertebral outlier) are then generated by an inner five-fold grouped cross-validation on the outer training set, so that the combiner never sees in-sample predictions. For computational tractability the Stage 2 regularisation strength was not itself re-tuned within this inner loop but fixed at the midpoint of the grid used elsewhere; because the value is fixed a priori rather than chosen from the data, this simplification cannot introduce optimistic bias, although a full inner-inner search might further improve Stage 2. The combiner is fitted by logistic regression on the resulting two-dimensional inner-out-of-fold score vector, and its decision threshold is selected from out-of-fold predictions of the combiner itself. Once the combiner has been fixed, both Stage 1 and Stage 2 are retrained on the full outer training set and the combined score is evaluated on the held-out patient $i^{*}$. The protocol guarantees that no information from $i^{*}$ enters any step.

### Appendix A.2. Pre-Quality-Control Cohort Counts

Pre-quality-control cohort counts (before excluding pre-existing fractures and the audit-level patient exclusions) were as follows. The abdominal CT cohort included 127 candidate patients with 635 candidate L1 to L5 vertebrae, of which 38 vertebrae were excluded as pre-existing fractures (all five vertebrae of one of those 127 patients were excluded for that reason, leaving 126 retained patients in Table 1) and eight as segmentation failures, giving the 589 evaluable vertebrae reported in Table 1. The lumbar CT cohort included 106 candidate patients with 530 candidate vertebrae, of which 36 were excluded as pre-existing fractures and 16 because of segmentation or reconstruction failures, giving the 478 evaluable vertebrae over 106 retained patients reported in Table 1.

## References

[1] Cosman F., de Beur S.J., LeBoff M.S., Lewiecki E.M., Tanner B., Randall S., Lindsay R. (2014). Clinician’s guide to prevention and treatment of osteoporosis. Osteoporos. Int..

[2] Johnell O., Kanis J.A. (2006). An estimate of the worldwide prevalence and disability associated with osteoporotic fractures. Osteoporos. Int..

[3] Ballane G., Cauley J.A., Luckey M.M., El-Hajj Fuleihan G. (2017). Worldwide prevalence and incidence of osteoporotic vertebral fractures. Osteoporos. Int..

[4] Lenchik L., Rogers L.F., Delmas P.D., Genant H.K. (2004). Diagnosis of osteoporotic vertebral fractures: Importance of recognition and description by radiologists. Am. J. Roentgenol..

[5] Buckens C.F., de Jong P.A., Mol C., Bakker E., Stallman H.P., Mali W.P., van der Graaf Y., Verkooijen H.M. (2013). Intra- and interobserver reliability and agreement of semiquantitative vertebral fracture assessment on chest computed tomography. PLoS ONE.

[6] Link T.M., Kazakia G.J. (2020). Update on imaging-based measurement of bone mineral density and quality. Curr. Rheumatol. Rep..

[7] Schousboe J.T. (2016). Epidemiology of vertebral fractures. J. Clin. Densitom..

[8] Buchbinder R., Johnston R.V., Rischin K.J., Homik J., Jones C.A., Golmohammadi K., Kallmes D.F. (2018). Percutaneous vertebroplasty for osteoporotic vertebral compression fracture. Cochrane Database Syst. Rev..

[9] Pickhardt P.J., Pooler B.D., Lauder T., del Rio A.M., Bruce R.J., Binkley N. (2013). Opportunistic screening for osteoporosis using abdominal computed tomography scans obtained for other indications. Ann. Intern. Med..

[10] Lee S.J., Pickhardt P.J. (2018). Future osteoporotic fracture risk related to lumbar vertebral trabecular attenuation at routine body CT. J. Bone Miner. Res..

[11] Boutin R.D., Lenchik L. (2020). Value-added opportunistic CT: Insights into osteoporosis and sarcopenia. Am. J. Roentgenol..

[12] Pickhardt P.J., Graffy P.M., Zea R., Lee S.J., Liu J., Sandfort V., Summers R.M. (2020). Automated abdominal CT imaging biomarkers for opportunistic prediction of future major osteoporotic fractures in asymptomatic adults. Radiology.

[13] Bodden J., Sollmann N., El Husseini M., Sekuboyina A., Löffler M.T., Zimmer C., Kirschke J.S., Baum T. (2023). Incidental vertebral fracture prediction using automatic spine segmentation and volumetric BMD extraction from routine clinical CT. Front. Endocrinol..

[14] Goller S.S., Reidler P., Rudolph J., Stahl R., Wulfff A., Gersing A.S. (2023). Automated opportunistic trabecular volumetric bone mineral density extraction outperforms manual measurements for the prediction of vertebral fractures in routine CT. Diagnostics.

[15] Isensee F., Jaeger P.F., Kohl S.A.A., Petersen J., Maier-Hein K.H. (2021). nnU-Net: A self-configuring method for deep-learning-based biomedical image segmentation. Nat. Methods.

[16] Wasserthal J., Breit H.-C., Meyer M.T., Pradella M., Hinck D., Sauter A.W., Heye T., Boll D.T., Cyriac J., Yang S. (2023). TotalSegmentator: Robust segmentation of 104 anatomic structures in CT images. Radiol. Artif. Intell..

[17] Allaire B.T., Lu D., Johannesdottir F., Kopperdahl D., Keaveny T.M., Jarraya M., Guermazi A., Bredella M.A., Samelson E.J., Kiel D.P. (2019). Prediction of incident vertebral fracture using CT-based finite element analysis. Osteoporos. Int..

[18] Kong S.H., Ahn D., Kim B., Srinivasan K., Ram S., Kim H., Hong A.R., Kim J.H., Cho N.H., Shin C.S. (2024). A CT-based fracture prediction model with images of vertebral bones and muscles by employing deep learning. J. Med. Internet Res..

[19] Kong S.H., Choi S., Cho W., Park S.B., Park S.S., Choo J., Kim J.H., Kim S.W., Shin C.S. (2025). Enhancing vertebral fracture prediction using multitask deep learning of CT imaging of bone and muscle. Eur. Radiol..

[20] Dieckmeyer M., Löffler M.T., El Husseini M., Sekuboyina A., Menze B., Sollmann N., Wostrack M., Zimmer C., Baum T., Kirschke J.S. (2022). Level-specific volumetric bone mineral density threshold values for prediction of incident vertebral fractures using opportunistic quantitative CT. Front. Endocrinol..

[21] Hummel J., Ferraro F., Engelke K., Chaudry O. (2025). Trabecular texture and paraspinal muscle characteristics for prediction of first vertebral fracture: A quantitative-CT analysis from the AGES cohort. Front. Endocrinol..

[22] Wang M., Wu Y., Hu Q., Wang J., Yang J., Ge Y., Yu Y., Tao X., Chen X. (2023). A CT-based radiomics nomogram for predicting osteoporotic vertebral fractures: A longitudinal study. J. Clin. Endocrinol. Metab..

[23] Zhang D., Wang J., Zhang Y., Wei Z., Wang Y., Tang H., Shen J., Chen X., Xie C. (2026). CT-based bone habitat radiomics for predicting risk of vertebral fracture in older adults: A longitudinal study. Glob. Spine J..

[24] Tang H., Zhang X., Yu Y., Hu Q., Wu Y., Yang J., Ge Y., Chen X. (2025). The association between computed-tomography-based osteosarcopenia and osteoporotic vertebral fractures: A longitudinal study. J. Endocrinol. Investig..

[25] Zhang J., Wang X., Liu J., Zhang L. (2024). Development and validation of a predictive model for vertebral fracture risk in osteoporosis patients. Eur. Spine J..

[26] Namireddy S.R., Gill S.S., Peerbhai A., Kamath A.G., Ramsay D.S.C., Ponniah H.S., Salih A., Jankovic D., Kalasauskas D., Neuhoff J. (2024). Artificial intelligence in risk prediction and diagnosis of vertebral fractures. Sci. Rep..

[27] Li Y., Liang Z., Li Y., Cao Y., Zhang H., Dong B. (2024). Machine-learning value in the diagnosis of vertebral fractures: A systematic review and meta-analysis. Eur. J. Radiol..

[28] Wang X., Ye W., Gu Y., Gao Y., Wang H., Zhou Y., Pan D., Ge X., Liu W., Cai W. (2025). Predicting secondary vertebral compression fracture after vertebral augmentation via CT-based machine-learning radiomics-clinical model. Acad. Radiol..

[29] Yang J., Zhang S.-B., Yang S., Ge X.-Y., Ren C.-X., Wang S.-J. (2025). CT-based radiomics predicts adjacent vertebral fracture after percutaneous vertebral augmentation. Eur. Spine J..

[30] Kim Y., Kim Y.G., Park J.W., Kim B.W., Shin Y., Kong S.H., Kim J.H., Lee Y.K., Kim S.W., Shin C.S. (2024). A CT-based deep-learning model for predicting subsequent fracture risk in hip-fracture patients. Radiology.

[31] Muehlematter U.J., Mannil M., Becker A.S., Vokinger K.N., Finkenstaedt T., Osterhoff G., Fischer M.A., Guggenberger R. (2019). Vertebral body insufficiency fractures: Detection of vertebrae at risk on standard CT images using texture analysis and machine learning. Eur. Radiol..

[32] Vabalas A., Gowen E., Poliakoff E., Casson A.J. (2019). Machine-learning algorithm validation with a limited sample size. PLoS ONE.

[33] Genant H.K., Wu C.Y., van Kuijk C., Nevitt M.C. (1993). Vertebral fracture assessment using a semiquantitative technique. J. Bone Miner. Res..

[34] Zwanenburg A., Vallières M., Abdalah M.A., Aerts H.J.W.L., Andrearczyk V., Apte A., Ashrafinia S., Bakas S., Beukinga R.J., Boellaard R. (2020). The Image Biomarker Standardization Initiative: Standardized quantitative radiomics for high-throughput image-based phenotyping. Radiology.

[35] Zhao F.D., Pollintine P., Hole B.D., Adams M.A., Dolan P. (2009). Vertebral fractures usually affect the cranial endplate because it is thinner and supported by less-dense trabecular bone. Bone.

[36] Sineglazov V.M., Riazanovskiy K.D., Chumachenko O.I. (2020). Multicriteria conditional optimization based on genetic algorithms. Syst. Res. Inf. Technol..

[37] van Griethuysen J.J.M., Fedorov A., Parmar C., Hosny A., Aucoin N., Narayan V., Beets-Tan R.G.H., Fillion-Robin J.-C., Pieper S., Aerts H.J.W.L. (2017). Computational radiomics system to decode the radiographic phenotype. Cancer Res..

[38] Sineglazov V.M., Riazanovskiy K.D., Klanovets A., Chumachenko O.I., Linnik N. (2022). Intelligent tuberculosis activity assessment system based on an ensemble of neural networks. Comput. Biol. Med..

[39] Zgurovsky M., Sineglazov V., Chumachenko E. (2021). Classification and Analysis Topologies of Known Artificial Neurons and Neural Networks. Stud. Comput. Intell..

[40] Chen Y., Che M., Yang H., Yu M., Yang Z., Qin J. (2025). Predicting thoracolumbar vertebral osteoporotic fractures: Value assessment of chest CT-based machine learning. Acad. Radiol..

[41] Coniglio A., Rava A., Fusini F., Zoccola K., Contò M., Massè A., Girardo M. (2021). Effectiveness and reliability of cannulated fenestrated screws augmented with polymethylmethacrylate cement in the surgical treatment of osteoporotic vertebral fractures. J. Craniovertebr. Junction Spine.

[42] Zuluaga-Garcia J.P., Sierra M.A., Call-Orellana F.A., Herrera D., Andrade-Almeida R.A., Ravindran P.K., Ramirez-Ferrer E. (2025). Complications of vertebroplasty in adults: Incidence, etiology, and therapeutic strategies—A comprehensive, systematic literature review. Complications.

[43] Rose L.D., Bateman G., Ahmed A. (2024). Clinical significance of cement leakage in kyphoplasty and vertebroplasty: A systematic review. Eur. Spine J..

[44] Wang H., Sribastav S.S., Ye F., Yang C., Wang J., Liu H., Zheng Z. (2018). Balloon kyphoplasty versus percutaneous vertebroplasty for osteoporotic vertebral compression fracture: A meta-analysis and systematic review. J. Orthop. Surg. Res..

